# Supplementary material for: Malignant Melanoma of the Tongue: A Scoping Review
Source: Life (Basel). 2025 Jan 28;15(2):191. doi: 10.3390/life15020191 (PMC11856353; doi:10.3390/life15020191)
Supplement: Supplementary file 1 [file life-15-00191-s001.zip › Table S1.pdf]

Table S1. Summary of demographic, clinical, and histological characteristics of 47 cases of tongue melanoma.

Cases/ Articles	M/F (n =43)	Age (years) (n = 40)	Site (n = 37)	Max diameter (cm) (n = 31)	Clinical morphology (n = 33)	Metastasis, local invasion and recurrences (at the time of diagnosis) (n = 39)	Histology (n = 23)
**47/47**	**25/18**<br>M: 58,14 %<br>F: 41,86%<br>NP: 4	**0-5**: 0 (0%)<br>**6-13**: 1 (2,5%)<br>**14-18**: 0 (0%)<br>**19-33:** 5 (12,5%)<br>**34-48**: 3 (7,5%)<br>**49-64:** 13 (32,5%)<br>**65-78**: 14 (35%)<br>**79 and over**: 4 (10%)<br>- **NP**: 7<br><br>Mean: 58,6<br>Median: 62<br>Range: 7–90	**Root**: 0 (0%)<br>**Body**: 35 (94,59%)<br>- Base: 11 (29,72%)<br>- Dorsum: 12 (32,43%)<br>- Lateral borders: 12 (32,43%)<br>**Apex**: 2 (5,4%)<br><br>**NP:** 10	- **≤ 0.5**: 1 (3,22%)<br>- **0.6- 0.9:** 2 (6,45%)<br>- **1.0-1.9**: 7 (22,58%)<br>- **2.0– 2.9:** 5 (16,12%)<br>- **3.0– 3.9:** 8 (25,8%)<br>- **≥ 4.0**: 8 (25,8%)<br><br>**Median diameter**: 2,69<br><br>**Range:** 0,4 – 6,2<br><br>**NP:** 16	- **Papule/Nodule:** 27 (81,81%)<br>- **Macule/Patch**: 3 (9,09%)<br>- **Submucosal mass**: 2 (6,06%)<br>- **Plaque**: 1 (3,03%)<br><br>**Secondary morphology** (records already included in previous classification):<br>- Black color: 21<br>- Ulceration and bleeding: 11<br>- Amelanotic: 6<br>- Multiple lesions: 3<br>- Satellitosis: 2<br>- Giant mass (>6 cm): 2<br>- Cystic appearance: 1<br>- Associated with leukoplakia: 1<br><br>- **NP**: 14	- **Distant metastasis:** 15 (38,46%)<br>Lung and pleura: 6<br>Brain: 4<br>Liver: 1<br>Spinal cord: 1<br>- **Nodal metastasis**: 14 (35,9%)<br>- **No metastasis**: 10 (25,64%)<br>- **Unknown staging (NP)**: 8<br><br>- **Local Recurrence**: 8 (20,51%)<br>- **Local invasion**: 5 (12,82%)	**Clear cell sarcoma:** 4 (17,39%)<br>**Epithelioid:** 3 (13,04%)<br>**Spindle Cells**: 8 (34,78%)<br>**Epithelioid and Spindle Cells:** 8 (34,78%)<br><br>-Infiltration of the underlying tissues or the presence of satellite nodules: 11/23<br>- Pagetoid spread: 3/23<br>**-** Vascular and/or perineural invasion: 4/23<br><br>**Immunohistochemistry** (n = 17):<br>- HMB45: 13/17<br>- S100: 11/17<br>- SOX- 10: 4/17<br>- MART-1/Melan-A: 3/17<br>- Vimentin: 3/17<br><br>**NP**: 24

Abbreviations: F, female; HMB-45, Human Melanoma Black 45; M, male; MART-1, Melanoma Antigen Recognized by T cells 1; n, records included in every column; NP, not published; SOX-10, SRY-box transcription factor 10.
