# Supplementary material for: Malignant Melanoma of the Tongue: A Scoping Review
Source: Life (Basel). 2025 Jan 28;15(2):191. doi: 10.3390/life15020191 (PMC11856353; doi:10.3390/life15020191)
Supplement: Supplementary file 1 [file life-15-00191-s001.zip › Table S2.pdf]

Table S2. List of publications included in the review.

First author	Year	Age	Sex	Site	Cases	Clinical Morphology	Time to treatment of Primary Lesion	Recurrence or Metastasis	Therapy	Histology
Baxter	1941	NP	NP	NP	1	NP	NP	NP	NP	NP
Blackburn	1951	90	F	Lateral aspect	1	Polypoid mass measur-ing 2.5 cm in diameter and appearing grayish black in color	NP	Local recurrence and metastatic disease	Unspecified surgical procedure	NP
Moore	1955	NP	M	NP	1	NP	NP	NP	NP	NP
Aponte	1956	7	F	Left lateral margin	1	Painless, rounded, saucer-like, nontender, sessile growth	1 months	No	Simple excision	Fusiform or spindle cells with prominent nuclei
Kragh	1960	NP	NP	NP	1	NP	NP	NP	NP	NP
Amoretti	1962	75	F	Ventral surface	1	Multilobular pedunculated black tumor measuring 3 X 2cm	6 months	Widespread metastasis	Simple excision	NP
Milton	1965	71	M	Right side	1	Large, black, polypoid, fungating and in places ulcerating mass	6 years	Locally invasive	WLE	Malignant melanoma
Principato	1965	74	M	Dorsum	1	Painless flat fairly soft black-gray 1 x 1.5 cm superficial plaque	NP	Lung and cervical nodal metastasis	WLE + RT	Polygonal and spindle shaped cells

Reali-Forster	1966	NP	NP	NP	1	NP	NP	NP	NP	NP
Kopec	1966	NP	F	NP	1	Arising in pregnancy	NP	NP	NP	NP
Catlin	1967	NP	NP	NP	1	NP	NP	NP	NP	NP
Trodahl	1970	69	M	NP	1	NP	NP	NP	NP	NP
Kumar	1972	53	M	Base	1	NP	NP	NP	NP	NP
Takagi	1974	NP	F	NP	1	NP	NP	NP	NP	NP
Lukács	1980	61	M	Dorsum	1	NP	NP	Metastatic disease	Immunotherapy	NP
Kalemeris	1985	47	M	NP	1	Following low dose of radiation therapy	NP	NP	Primary: WLE<br>Metastasis: CT	Spindle cells
Bovo	1996	57	M	Base	1	Heavily pigmented, rather bulky and nodular lesion, approximately 0.4 cm in size,	NP	Brain and lungs	Unspecified surgical procedure	NP
Tanaka	1997	62	F	Left posteriolateral side	1	Black, pigmented, ulcerated mass measuring approximately 4.7 cm in diameter with extension to epiglottis	12 months	NP	Palliative chemotherapy	Parakeratinizing stratified squamous epithelium that exhibited an area of ulceration and melanotic

										junctional activity; HMB-45 +
Folz	1998	87	M	Left base	1	NP	NP	NP	Laser Co2 (palliative therapy)	NP
Spiegel	1999	65	F	Right dorsal surface	1	Raised mass from within the pigmented region	NP	NP	None (for patient's decision)	NP
Misawa	2000	65	M	Base	1	Nodular irregular mass	NP	Metastatic	Primary: WLE + immunotherapy; Metastasis: radical neck dissection + chemohormone therapy, immunotherapy	NP
Tanaka	2001	62	F	Dorsum	1	NP	NP	Metastatic	Surgery + CT	NP
Chiu	2002	66	F	Right lateral border	1	Black, pigmented and ulcerated mass	7 years	No	WLE + functional neck dissection	Epithelioid to spindle melanocytes with skeletal muscle invasion
Rowland	2003	53	M	Midline of the dorsum	1	8 mm pale mass (amelanotic)	5 years	Lungs and brain	Primary: WLE; Metastasis: RT	Epithelioid malignant melanoma, with associated pagetoid spread; HMB-45 +, S-100 +
Garzino-Demo	2004	72	F	Dorsum	1	Pigmented nodular	NP	Locally invasive	WLE + neck dissection	NP
Chikumaru	2008	69	M	Dorsum	1	1.5 × 1.5 × 0.8 cm pedunculated, grey-black, pigmented nodule in size.	3 months	No	Palliative therapy (cyclophosphamide and interferon alfa-2b)	Epithelial-like cells, including numerous atypical melanin pigment cells; HMB-45 +, S-100 +

Kaehler	2008	57	M	Right side of the tongue	1	Brownish black pigmented lesion	9 years	Yes (unspecified)	Simple excision + interferon alfa-2b	Invasive spindle cell desmoplastic melanoma; HMB-45 +, S-100 +
Khalifa	2009	72	F	Right postrolateral aspect	1	Black pigmented ulcerated mass measuring about 3 × 2 cm	6 months	No	WLE + neck dissection	Proliferation of epithelioid to spindle melanocytes, with skeletal muscle invasion
Zimmermann	2011	66	M	Base	1	3 cm exophytically pedunculated, partially bluish mass	NP	Local recurrence and lymph nodes metastasis	WLE + interferon alfa-2b	Epithelioid to spindle melanocytes; positive for MART1/Melan A, HMB-45 e S-100
Lee	2013	49	M	Lateral border	1	Slightly elevated black tumor with a smooth surface	3 months	No	WLE with neck dissection + RT + CT	Local invasive, HMB-45 +, S-100 + (15% of cells)
Venugopal	2013	19	M	Left lateral border	1	Raised non-tender lesion on the tongue	NP	Cervical lymph nodes	WLE with neck dissection	Spindle shaped and also epitheloid with nuclear pleomorphism, prominent nucleoli, and tumor giant cell formations; HMB-45 +, S-100 +
Kraft	2013	82	M	Body	1	Progressing tongue lesion and also a left-sided neck mass.	12 months	Cervical and thoracic lymph nodes	Total glossectomy with neck dissection + RT	neoplasm composed of sheets, nests, and fascicles of epithelioid and spindle cells in a fibrocollagenous stroma (clear cell sarcoma); HMB-45 -, S100 +
Kumar	2013	50	F	Right lateral margin	1	5 × 5 cm ulceroproliferative growth (amelanotic) on a site of a prior squamous cell carcinoma	12 months	Local recurrence and brain metastases	WLE + interferon alfa-2b	Malignant melanoma with spindle cells; HMB45+, S100 -, vimentin -.
Rubio-Correa	2014	51	M	Base	1	Black, pigmented and diffuse mass measuring approximately 3 X 3 cm in size	NP	No	Subtotal glossectomy + interferon alfa-2b	NP

Singh	2014	29	F	Tip	1	NP	2 months	No	WLE	Nests and fascicles of predominantly spindle-shaped cells with a vesicular nucleus and perineural invasion (clear cell sarcoma); HMB-45 +, S-100 +
Yang	2015	54	M	Left side of the base	1	Cystic black lesion	2 months	Pleura, lungs and mediastinal lymph nodes	SE	Malignant melanoma, HMB-45 +, S-100 +, vimentin +.
Alkaff	2017	73	M	Right lateral portion of the mobile tongue	1	4 × 4 cm mass with an ulcer with invasion of gingiva	3 months	Lung	WLE + neck dissection	Primary malignant melanoma
Abu-Zaid	2018	30	M	Dorsum	1	Numerous, flat to slightly raised, ill-defined, non-tender and purple lesions involving the dorsum of the tongue with scattered islands of leucoplakia	2 months	No	Unspecified surgical procedure	Epithelioid and spindle-shaped malignant melanocytes; HMB-45 +, S-100 +
Mayo	2019	63	F	Left posterior tongue	1	White, tender, 1-cm nodule (following skin graft reconstruction) with pigmented satellites of the left base of tongue, pharyngeal wall, and piriform sinus	1 month	Cervical lymph nodes and brain metastases	Lateral tongue excision with biopsy of the satellite lesions + pembrolizumab	Atypical epithelioid cells harboring pigmen, SOX-10 +.
Baus	2019	44	F	Ventral surface	1	Well-limited and hard tumefaction without mucosal lesions.	2 years	No	Neo-adjuvant CT + WLE + adjuvant RT	Malignant proliferation of clears cells (CCS), positive for P100 and Bcl2 markers, CK7, CD34
Swain	2021	68	M	Right side of the base	1	2.5 x 3 cm black-colored mass	6 months	No	WLE	Epithelial-to-spindle melanocytes along with a deposition of melanin

Motiee-Langroudi	2021	33	F	Left posterolateral part	1	1 cm pigmented ulcerated mass with a satellite smaller discoloration	5 months	Local recurrence	Primary: WLE + imatinib; Recurrence: Surgery + RT	Malignant melanoma with satellite lesions with infiltration of muscles and lymphovascular invasion; c-KIT +
Soares	2021	77	M	NP	1	NP	NP	NP	Surgery + CT	NP
Leite	2021	62	M	Distal extremity	1	2 cm non-pigmented tumor (amelanotic)	5 months	Lymph nodes, lung and liver metastases	Primary: partial glossectomy Metastasis: elective node dissection + RT	Mucosal melanoma with ulceration; S100 +; MART1 +, vimentin +
Seghrouchni	2023	88	M	Right border	1	Large, focally ulcerated nodular lesion	3 months	No	WLE + RT	Nested proliferation of large, epithelioid, clear, cells with perineural invasion (clear cell sarcoma); S100 +, HMB-45 +, SOX10 +, and vimentin +
Arora	2024	46	F	Left side of tongue	1	Black colored patch	2 months	Cervical lymph node	Partial glossectomy + left sided selective neck dissection	Solid sheets of tumor cells, with pagetoid spread and lymphovascular invasion; HMB-45 +; SOX-10 +
Kumar	2024	27	M	Dorsum	1	Large, painless, ulceroproliferative mass (amelanotic melanoma)	6 months	Cervical lymph nodes metastasis	subtotal glossectomy with bilateral neck lymph node dissection	epithelioid to spindle cells displaying moderate nuclear pleomorphism, large prominent nucleoli, with high mitotic activity; HMB-45 +, S-100 +

Abbreviations: CT, chemotherapy; F, female; HMB-45, Human Melanoma Black 45; M, male; MART-1, Melanoma Antigen Recognized by T cells 1; NP, not published; RT, radiation therapy; WLE, wide local excision; SOX-10, SRY-box transcription factor 10.
